# Metagenomic binning of PacBio HiFi data prior to assembly reveals a complete genome of *Cosmopolites sordidus* (Germar) (Coleopterea: Curculionidae, Dryophthorinae) the most damaging arthropod pest of bananas and plantains

**DOI:** 10.7717/peerj.16276

**Published:** 2023-11-22

**Authors:** Alfredo Rodriguez Ruiz, Alex R. Van Dam

**Affiliations:** Departamento de Biología, Universidad de Puerto Rico Recinto Universitario de Mayagüez, Mayagüez, Puerto Rico, United States of America

**Keywords:** Metagenomics, HiFi assembly, Insect genome assembly, Metagenomic binning

## Abstract

PacBio HiFi sequencing was employed in combination with metagenomic binning to produce a high-quality reference genome of *Cosmopolites sordidus*. We compared k-mer and alignment reference based pre-binning and post-binning approaches to remove contamination. We were also interested to know if the post-binning approach had interspersed bacterial contamination within intragenic regions of Arthropoda binned contigs. Our analyses identified 3,433 genes that were composed with reads identified as of putative bacterial origins. The pre-binning approach yielded a *C. sordidus* genome of 1.07 Gb genome composed of 3,089 contigs with 98.6% and 97.1% complete and single copy genome and protein BUSCO scores respectively. In this article we demonstrate that in this case the pre-binning approach does not sacrifice assembly quality for more stringent metagenomic filtering. We also determine post-binning allows for increased intragenic contamination increased with increasing coverage, but the frequency of gene contamination increased with lower coverage. Future work should focus on developing reference free pre-binning approaches for HiFi reads produced from eukaryotic based metagenomic samples.

## Introduction

The banana weevil, *i.e*., *Cosmopolites sordidus* (Germar) (Coleoptera: Curculionidae) is an economically important pest that has infested the banana and plantain industry worldwide (Gold, Pena & Karamura, 2001). *C. sordidus* is the most damaging arthropod pest of banana and plantain agriculture globally (Gold & Tinzaara, 2008). Bananas and plantains (*Musa spp*.) are amongst the world’s most valuable agricultural crops in developing countries (Ploetz et al., 2015). They also serve as a food staple and contribute significantly to food security in many low income food-deficit countries (FAO, 2022). They are important because they are rated as the fourth most valuable food after rice, wheat and milk (Okolle et al., 2020). Damage caused by *C. sordidus* occurs when the larvae bore into the root vascular tissue of the banana corm, reducing nutrient and water uptake and weakening the stability of the plant (Alpizar et al., 2012). *C. sordidus* is also one of the few K-selected weevils laying a single egg with each oviposition event and living up to four years as an adult (Gold & Messiaen, 2000). According to Gold, Pena & Karamura (2001) *C. sordidus* attacks on newly planted banana stands can lead to crop failure, and weevil damage can result in plant loss, reduced bunch weights, mat disappearance and shortened banana stand life.

Even though they are cultivated mainly in backyard gardens, smallholder farms, and commercial plantations which serve as a source of employment to thousands of people and it is of agricultural importance worldwide, *Musa *spp. is a victim of constrained biotic factors such as pests (aside *C. sordidus*) and diseases (Okolle et al., 2020). To this day no single control strategy provides complete control for *C. sordidus* on bananas. Only a broad integrated pest management (IPM) approach, by combining an array of methods (Gold & Messiaen, 2000; Ocimati et al., 2016; Twesigye et al., 2018). Although the combination of these recommended IPM systems proves to be slightly effective in reducing the banana weevil population, they tend to be costly and often unaffordable for small growers that produce bananas and plantains for local markets and thus they are not widely adopted by farmers (Tresson et al., 2021).

Despite the extensive and recent chemical, physiological and transcriptomic studies (Valencia et al., 2016) there is no whole genome resource for *C. sordidus*. One of the difficulties in sequencing and assembly of weevil genomes is the relatively high percentage of repeat regions (Dias et al., 2021; Parisot et al., 2021; Van Dam et al., 2021). Repeat regions make the genome assembly process *via* short reads difficult and often result in a lack of resolution around such repeat regions (Hon et al., 2020; Cheng et al., 2021). Adding to the difficulty is the relatively large size of more than a gigabase of some recent weevil genome assemblies further confounding long stretches of repeat regions (Dias et al., 2021; Parisot et al., 2021; Van Dam et al., 2021). PacBio HiFi sequencing performs well with genomic regions of high GC contents and long repeat regions (Hon et al., 2020; PacBio, 2021). PacBio long-read and HiFi reads have also shown utility in producing high-quality assemblies of complex metagenomic samples (Pearman, Freed & Silander, 2020; Xie et al., 2020; Priest et al., 2021). The utility of PacBio HiFi long reads would allow us to span these repetitive elements to build and provide a high-quality genome assembly. Here we aim to use long-read PacBio HiFi data to develop a high-quality genome of *C. sordidus* for future genomic research and perhaps provide useful information for a more efficient and cost-effective method to deal with this destructive pest.

Two classes of metagenomic binning approaches currently exist, reference-based and reference free within each of those are direct alignment based approaches and k-mer frequency-based approaches (Cornet & Baurain, 2022; Ibañez-Lligoña et al., 2023). Many of the most recent assemblies that utilize HiFi data use a reference based or hybrid approach using BlobTools (Kumar et al., 2013; Laetsch & Blaxter, 2017; Challis et al., 2020; Childers et al., 2021; The Darwin Tree of Life Project Consortium et al., 2022). Kraken2 has also been shown to be quite valuable at binning long read data and provides a different reference based approach that utilizes k-mer frequencies to classify reads and contigs of mixed samples containing Bacteria and Curculionidae genomes (Wood, Lu & Langmead, 2019; Cornet & Baurain, 2022; Van Dam et al., 2022; Ibañez-Lligoña et al., 2023). Other recent metagenomic binning techniques require multiple libraries to build robust k-mer frequencies (Alneberg et al., 2014; Kang et al., 2015), and the current state-of-the-art for processing short read data utilizes machine learning to infer genomic bins (Nissen et al., 2021; Piera Lindez et al., 2023). A recent work has been specifically designed to bin long read data using machine learning however it has only been validated on Bacteria (Wickramarachchi & Lin, 2022). While *LRBinner* represents the current state-of-the-art for long read binning (Wickramarachchi & Lin, 2022) in this project we wanted to be a bit conservative and use the more widely used k-mer frequency reference based approach (Wood, Lu & Langmead, 2019) and reference alignment based approach (Laetsch & Blaxter, 2017) for binning HiFi data and contigs.

PacBio HiFi sequencing has been used effectively with a post-assembly or post-binning approach on hundreds of arthropod genomes through the Ag100Pest and Darwin Tree of Life projects (Childers et al., 2021; The Darwin Tree of Life Project Consortium et al., 2022). However, the utility of a pre-binning approach at metagenomic filtering of HiFi reads in arthropod genome assembly has received much less attention. Here we also aim to find out if pre-binning and post-binning approaches can produce a roughly comparable *C. sordidus* assembly using HiFi data. We specifically aim to identify if potential bacterial contamination is present in intragenic regions of the *C. sordidus* post-binning genome assembly.

## Materials and Methods

### Collection locality

Collection occurred at the Universidad de Puerto Rico Mayagüez, Estación Experimental Agrícola Isabela on February 26, 2021. Specimens of *Cosmopolites sordidus* were collected from a fallow field of banana plants (*Musa acuminata*). *C. sordidus* specimens were extracted from corms and stems of banana pups. Several fully grown adults and one pharate adult were collected. Specimens were washed with distilled water before extraction on March 2, 2021. The individual that was initially collected as a pupa was now a pharate adult on the day of the DNA extraction.

### DNA extraction

DNA was extracted at the UPRM Invertebrate Collection Molecular Lab using the Circulomics Nanobind CBB Big DNA Arthropod Kit, (Part#NB-900-001-01; Circulomics, Baltimore, MD, USA) using the arthropod “Harvestman” protocol. The entire body of the pharate adult was used for the other adult specimens only legs, gonads, and thoracic muscle tissue was added to the extraction buffer. Using a disposable 1.5 ml Corning micro-pestle the *C. sordidus* tissue was crushed directly in the extraction buffer. Following the Nanobind DNA extraction, a single round of AMPure XP (Beckman Coulter, Inc, Brea, CA, USA) bead cleanup using a 0.8x dilution of beads to DNA extraction volume. This was done to eliminate as much carry over proteins as possible. Final volume was 40 µl at greater than 200 ng/µl as measured on a Promega Quantus Fluorometer. To confirm that the extracted DNA was of high molecular weight (HMW), we conducted agarose electrophoresis gel.

### HiFi library preparation and sequencing

The DNA extractions were stored at −15 °C and then shipped to the University of Maryland Genome School of Medicine, Genomics Resource Center on March 8, 2021. Samples were inspected for HMW quality and quantity *via* Agilent Technologies 5,200 Fragment Analyzer System (Agilent Technologies, Santa Clara, CA, USA). After inspection we decided only to carry forward the DNA extraction from the pharate adult. It had a main peak of 47,834 bp at 79.8% of the relative concentration, with a bell-shaped peak ranging from 500–134,830 bp with an average fragment size of 33,965 bp. The total DNA extraction volume and weight of DNA were determined to be 39.0 µl and 10,062.0 ng. A standard PacBio HiFi library preparation kit (PacBio, Menlo Park, CA, USA) aimed at 10–20 KB circular consensus sequence with the desired insert size around 15 KB was performed. Sequencing was carried out using a single SMRT Cell *via* a PacBio Sequel II, *via* sequencing run of a 30-h movie.

### Overall bioinformatics strategy

To measure the effect of pre- *vs* post-metagenomic binning to reference genomes we took the *C. sordidus* HiFi reads before assembly (pre-binning) and mapped them to the same reference database as the contigs will be in the post-binning strategy. Then both assemblies were repeat masked using a custom model developed from the prefiltered assembly. Next individual genome gene prediction analyses followed each individually masked assembly. Finally, each set of predicted proteins was annotated to various protein databases. For a graphical representation see Fig. S1 for a summary of the overall approach. The effect on gene prediction and annotation was then quantified. Any putative contamination in the post-binning strategy was cross-referenced against the reads with taxonomic annotations from the pre-binning strategy. Below is a detailed step-by-step breakdown.

### HiFi read processing

PacBio CCS v.6.6.3 (PacBio, 2021) software processed the PacBio data from the Sequel II HiFi library sequencing. Following the completion of the circular consensus sequences (ccs) bamtools v-2.5.1 was used to convert the ccs reads into HiFi reads *via* the command ‘-tag “rq”:“>=0.99”’ (Barnett et al., 2011). Next we removed putative adapter contamination *via* HiFiAdapterFilt with default settings, 44 bp match at 97% identity (Sim et al., 2022). We then used these HiFi reads as input for the assembly program hifiasm version 0.15.1-r334 (Cheng et al., 2021).

### Initial HiFi assembly, contig, and HiFi read metagenomic binning

#### Assembly program

Hifiasm was chosen because it tends to give longer assembled contigs than other HiFi assembly programs and does well at purging duplicated regions in heterozygous genomes (Cheng et al., 2021). We used hifiasm default parameters and 32 threads on the Extended Memory node of Bridges2 located at the Pittsburgh Supercomputing Facility to do the initial HiFi assembly.

#### Custom reference genome database

The rice weevil, red palm weevil, carrot weevil and easter egg weevil were used as arthropod representatives. The *Musa acuminiata* genome was used to help filter out any plant contamination. Representative microbial eukaryote genomes such as fungi, nematodes and representative protists were also included. A complete list of the eukaryotic genomes and their accession numbers can be found in Table S1. Bacterial genomes were added to the reference database *via* the Kraken2 Bacteria database. The reference genomes used if not already soft masked for repetitive elements were then masked using Dustmasker (Morgulis et al., 2006) implemented *via* blast tools v-2.11.0 (Camacho et al., 2009).

#### Metagenomic binning approach

Previous work on weevil genomes has shown substantial amounts of bacterial and symbiotic microbial eukaryote contamination can occur even with “clean” samples (Van Dam et al., 2022). The utility of Hi-C libraries would be optimal to physically link separate contigs into larger scaffolds or chromosomes as in the easter egg weevil genome (Van Dam et al., 2021) however Hi-C libraries for this project were not available. On the other hand, Hi-C assembly requires that the HiFi or long reads are first assembled separately and then scaffolded with Hi-C data. It remains unclear if bacterial contamination is incorporated in HiFi assemblies resulting in chimeric scaffolds. To see if there is any measurable effect of putative bacterial, microbial eukaryote, or host plant contamination in our assembly we decided to both pre-bin the HiFi reads and post-bin the HiFi based contigs. Kraken2 and BlobTools were used to bin HiFi reads to a custom database where only reads that were classified to Arthropoda would be passed onto the assembly step. After the assembly process, the post-binning strategy would filter out Arthropoda to the same custom database.

#### Post-binning strategy

To eliminate contaminant contigs from downstream analyses during the gene prediction step, two approaches were used to classify and assess the level of contamination in the initial hifiasm assembly. We took two approaches: an alignment-based approach and a k-mer alignment-free approach to bin contigs to phylum.

An alignment-based approach is widely used for metagenomic binning of a single library per sample is to leverage scaffold coverage cutoff, GC content, and annotating the scaffolds *via* a reference database (Laetsch & Blaxter, 2017; Leidenfrost et al., 2020). For an alignment-based approach we used the BlobTools pipeline (Laetsch & Blaxter, 2017) which employs blast+ (Camacho et al., 2009) to a custom genomic database for taxonomic annotation *via* blast v.2.11.0, coverage and GC content to inform metagenomic binning of assembly taxonomic annotation *via* (Kumar et al., 2013; Laetsch & Blaxter, 2017). Minimap2 v 2.22-r1101 (Li, 2018) and samtools v-1.13.0 (Li & Durbin, 2009) was used to generate read coverage of initial hifiasm contigs. Blast+ settings were blastn-task megablast with a stringent e-value cutoff of 1e−50 and max_target_seqs set to 1. As long contigs receive multiple hits taxonomy of contigs is then assigned by “bestsum” rule *via* BlobTools taxify where each blast result is summed by score and the counts of other taxonomic annotations relative to each summed score by taxon across a query contig (Laetsch & Blaxter, 2017). The taxon with the best overall score across the query contig is then assigned that taxonomy (Laetsch & Blaxter, 2017).

The alignment-free k-mer based approach used Kraken2 (Wood, Lu & Langmead, 2019) to bin contigs to the same custom reference genomic database as before. Kraken2 utilizes a taxonomic annotation algorithm with greatest number of k-mer hits shared between the contig query and reference database to assign taxonomy to the lowest common ancestor of the contig (Wood, Lu & Langmead, 2019). Kraken2 v 2.1.2 was used with default settings.

The contig taxonomic annotation to Arthropoda from BlobTools and Kraken2 were then compared *via* custom *awk* scripts, and taxonomic annotation to the phylum Arthropoda was used to assign taxonomy of contigs belonging to the *Cosmopolites sordidus* genome. Only contigs that both methods classified to Arthropoda or if one method failed to classify but the other method classified as Arthropoda were passed onto the next bioinformatic step.

#### Pre-binning strategy

Using the same parameters as above we used *blastn* and the “bestsum” rule as above from BlobTools taxify and Kraken2 to classify the HiFi reads. Again, only reads that were classified as Arthropoda by both methods or were unclassified by either method but were classified as Arthropoda by a single method were retained before assembly. Using these Arthropoda classified reads we used hifiasm using the same parameters as above to produce our pre-binning *C. sordidus* assembly.

### Genome assembly completeness assessment

To compare the relative overlap of the taxonomy annotation a simple Venn diagram was plotted in R (R Core Team, 2021). Completeness of genome assembly was quantified *via BUSCO v 5.2.2* using the Insecta Odb 10 database (Seppey, Manni & Zdobnov, 2019; Kriventseva et al., 2019). Summary statistics were computed using BBMap stats.sh script (Bushnell, 2015). BlobTools gives an assessment of coverage per contig and GC content per contig. The initial assembly prior to either -binning method the k-mer abundance was evaluated *via* jellyfish 2.3.0-3 (Marçais & Kingsford, 2011) and uploaded to GenomeScope (Vurture et al., 2017) for a graphical estimate. Hfiasm also gives a graphical measure of coverage that was visually inspected for each assembly.

### Repeat masking

Based on evidence from other weevil genomes this species likely would also contain a substantial amount of repetitive DNA elements (Dias et al., 2021; Parisot et al., 2021; Van Dam et al., 2021). To reduce as many false matches as possible we attempted an initial *de novo* repeat masking using RepeatModeler2 v-2.0.1 (Flynn et al., 2020) using the NCBI reference database. This repeat model was developed using the pre-binning *C. sordidus* genome and then applied to both the pre-and post-binning *C. sordidus* genomes by RepeatMasker v-4.1.0 (Smit, Hubley & Green, 2015) to mask transposable elements in the Arthropoda filtered assemblies.

### Gene prediction

Softmasked Arthropoda contigs from both the pre- and post-binning approaches were each passed separately downstream to the gene prediction steps. Gene prediction was performed using Braker2 v 2.1.6 (Hoff et al., 2019; Brůna et al., 2021) to create a *de novo* gene model and concomitant gene prediction for *C. sordidus*. We attempted to map both the 454 *C. sordidus* RNA-seq data (NCBI:SRA: SRR2133845) *via* hisat2 v 2.2.1 (Kim et al., 2019) and minimap2 v 2.22-r1101 (Li, 2018) and the predicted proteins from the rice weevil genome (NCBI: RefSeq: GCF_002938485.1_Soryzae_2.0_protein.faa) *via* GenomeThreader v 1.7.1, and pass them to braker.pl as hints. However too few alignments were produced for GeneMark-EP (Brůna, Lomsadze & Borodovsky, 2020) and it kept failing at this stage in the Braker2 pipeline. So we decided to only use proteins from the Insecta Odb10 database as hints for the Braker2 pipeline *via* GenomeThreader (Gremme et al., 2005) to do the alignment as part of the pipeline. We decided to use Braker2 to run AUGUSTUS 3.4.0 (Stanke et al., 2006; Keller et al., 2011) to predict genes. AUGUSTUS performs well with a few input protein hints, but requires high-quality annotation, as is the case with the well-defined gene set (Stanke et al., 2006; Keller et al., 2011) from the Insecta Odb10 database (Kriventseva et al., 2019). This worked successfully and Braker2 was able to complete the gene prediction process. Gene prediction completeness was evaluated using BUSCO v 5.2.2 (Seppey, Manni & Zdobnov, 2019) run in protein mode on the resulting genes.

### Functional gene annotation

Annotation was carried out *via* the EggNog v 5.0 database (Huerta-Cepas et al., 2019). EggNog database assignment was completed *via* eggNOG-mapper v 2.1.7 (Cantalapiedra et al., 2021) using *DIAMOND v2.1.6* as the alignment tool (Buchfink, Xie & Huson, 2015; Buchfink, Reuter & Drost, 2021). Results from eggNOG-mapper were parsed using a series of custom Python and awk scripts and R Bioconductor PloGO2 scripts to generic cellular component GO-slims (Pascovici & Wu, 2021). Gene orthology of predicted proteins was also evaluated *via Orthofinder v 2.5.4* (Emms & Kelly, 2015, 2019) using the NCBI RefSeq (O’Leary et al., 2016) protein sets of *Drosophila melanogaster* (GCF_000001215.4_Release_6_plus_ISO1_MT), *Tribolium castaneum* (GCF_000002335.3_Tcas5.2), *Dendroctonus ponderosae* (GCF_000355655.1_DendPond_male_1.0), *Sitophilus oryzae* (GCF_002938485.1_Soryzae_2.0), and the predicted proteins for *C. sordidus*. Results were then visualized using *R* package Venn.Diagram (Chen & Boutros, 2011) and a custom Python script using venn, pandas and matplotlib (Hunter, 2007; McKinney, 2010; The Python Venn Developers, 2022).

### Identifying *C. sordidus* coding regions contaminated with HiFi reads of bacterial origins

First, the reads annotated as Bacteria from Kraken2 above were mapped to the post-binning genome assembly to see if any regions that were similar to the contamination existed. We used Minimap2 map-hifi to map and samtools to remove duplicate reads. We then filtered those reads from the SAM file to retain only the highest mapping quality of mapq20 or greater.

Next, we identified if the putative Kraken2 bacterial reads ended up in the assembly process by identifying their positions in the post-binning Hifiasm gfa file. We then used bedtools intersect (Quinlan & Hall, 2010) to identify the union of the read positions in the gfa file, the *Minimap2* results, and the gff file from braker2 to identify if apparent contamination was used in the assembly of genome coding regions. Custom Python scripts were used to graph these putative regions using histograms with and without overlap lengths *via* histogram smoothing using Kernel Density Estimation (KDE) *via* seaborn, numpy, and matplotlib (Virtanen et al., 2020; Harris et al., 2020; Waskom, 2021).

We also sorted each gene by unique overlaps using *awk* and *bash* followed by a custom Python script to calculate the average unique overlapping regions per gene. This was followed by a similar approach to find the average coverage of putative Bacteria integration per gene. We then visualize these results using a hexbin plot followed by a linear regression and logistic regression analyses using custom Python scripts using libraries matplotlib, and scipy (Virtanen et al., 2020).

### Final metagenomic contaminant classification *via* NCBI *FCS-GX*

The new metagenomic classification program supplied by NCBI, FCS-GX (Astashyn et al., 2023) was carried out on both post- and pre-binning assemblies as a final pass to identify any additional sources of contamination using the NCBI Genome submission portal. *FCS-GX* utilizes a very large database including assemblies from NCBI GenBank and RefSeq genomes, and acts as a redundant k-mer alignment-based post-filtering approach for both genomes (Astashyn et al., 2023).

### mtDNA genome annotation

CCS reads were aligned to the *Sitophilus oryzae* mtDNA genome (NCBI: NC_030765.1) using minimap2. The matching reads were then assembled *via* RagTag v 2.1.0 (Alonge et al., 2021) using the scaffold setting so that the resulting assembly would not incorporate the *S. oryzae* DNA into the final assembly (Alonge et al., 2021). The resulting assembly was then mapped back to the hifiasm contigs *via* blat v-36 (Kent, 2002), this resulted in only a handful of matches but no matches that were shorter than 20 KB. This made it ambiguous if the matching contigs were genuine mitochondrial DNA-like sequences in the nucleus (NUMTs) (Mishmar et al., 2004) or misassembled contigs. We decided to leave them in the final assembly for now but provide a psl file of the handful of matches if others decide they do not want to search against them. Finally, the predicted cox1 gene was for the putative mtDNA genome was aligned against the NCBI nucleotide database *via* the NCBI blastn web portal to ensure it was from *C. sordidus* and not another species. MitoFinder v 1.4 (Allio et al., 2020) was also used to annotate any putative mtDNA sequences in the NCBI genome submission.

## Results

### PacBio HiFi sequencing

PacBio HiFi Sequel II sequencing and yielded 27,928,246 subreads totaling 1,709,178 circular consensus sequences. There were 291 HiFi reads that contained adapters, or approximately 0.01% of the data. Adapter removal and quality filtering produced 1,708,887 HiFi reads, for full summary statistics produced by CCS and bamtools filtering of the reads can be found in Table 1.

**Table 1:** Sequencing results and statistics of the reads in the *Cosmopolites sordidus* genome sequencing project.

	Sub-reads
Bases	384,703,897,181
Reads	27,928,246
Mean	13,774.725
Median ± Stddev	14,967.000 ± 8,614.1055
Mode	15,196
min/max	50/527,147
p90/p95/p99	16,921/17,648/34,819
N50	15,450
	**CCS reads**
Bases	25,813,370,147
Reads	1,709,178
Mean	15,102.80
Median ± Stddev	15,067.000 ± 1,069.8483
Mode	14,798
min/max	45/33,123
p90/p95/p99	16,398/16,731/17,335
N50	15,134
	**CCS HiFi reads**
Bases	25,812,386,317
Reads	1,708,887
Mean	15,104.794
Median ± Stddev	14,493 ± 1,114,729.987
Mode	14,798
min/max	45/33,123
p90/p95/p99	16,398/16,731/17,335
N50	15,134

### Hifiasm assembly

**Figure 1:**
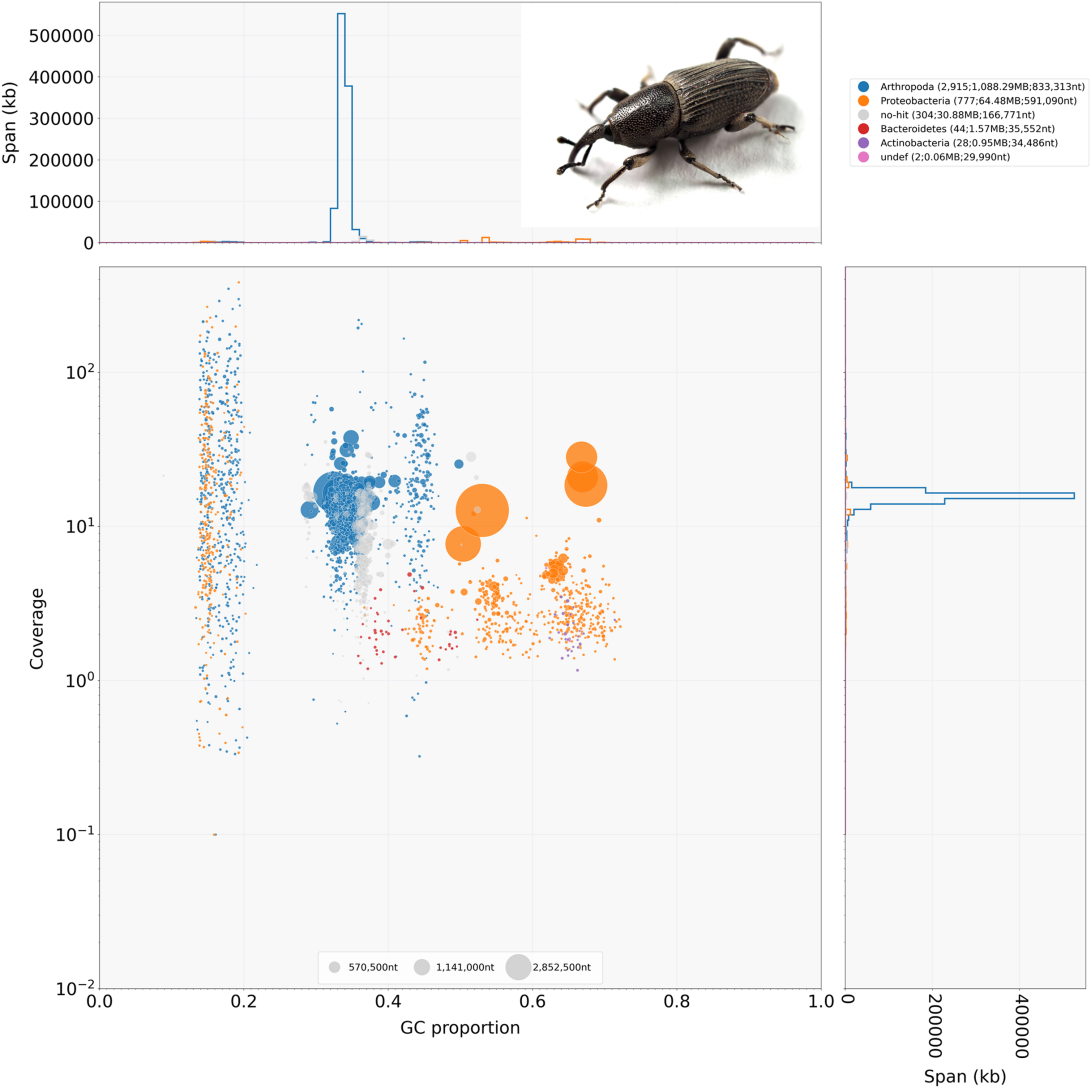
*BlobTools* blob plot of the initial *Cosmopolities sordidus* genome assembly *via* hifiasm. *Cosmopolites sordidus* habitus in the top right hand corner.

The initial assembly contained 4,070 contigs with a total length of 1,186.227 MB (1,186,226,763 bp) with 95% of the contigs >50 KB in length and an N/L50 of 412/811.698 KB, for a complete summary see Table 2. The pre- and post-binning assemblies each 3,089 and 3,076 contigs with 97% of the contigs >50 KB and an N/L50 of 444/703.967 KB and 395/824.959 KB respectively, for a full summary, see Table 2. Hifiasm reported the haploid coverage of 22X for both the pre- and post-binning genome assemblies. BlobTools gave an approximate coverage of about 20X for the post-binning genome assembly (Fig. 1).

**Table 2:** *Cosmopolites sordidus* initial *hifiasm* assembly, post-metagenomic binning assembly, and pre-metagenomic binning assembly summary statistics.

	Initial HiFiasm assembly	Post-metagenomic binning	Pre-metagenomic binning
Main genome contig total:	4,070	3,076	3,089
Main genome scaffold sequence total:	1,186.227 MB	1,103.270 MB	1,071.278 MB
Main genome contig sequence total:	1,186.227 MB 0.000% gap	1,103.270 MB 0.000% gap	1,071.278 MB 0.000% gap
Main genome scaffold N/L50:	412/811.698 KB	395/824.959 KB	444/703.967 KB
Main genome contig N/L50:	412/811.698 KB	395/824.959 KB	444/703.967 KB
Main genome scaffold N/L90:	1,575/154.206 KB	1,375/205.863 KB	1,571/178.152 KB
Main genome contig N/L90:	1,575/154.206 KB	1,375/205.863 KB	1,571/178.152 KB
Max scaffold length:	11.41 MB	6.71 MB	4.439 MB
Max contig length:	11.41 MB	6.71 MB	4.439 MB
Number of scaffolds >50 KB:	2,261	2,078	2,375
% main genome in scaffolds >50 KB:	95.36%	97.28%	97.96%

### Metagenomic binning

#### Post-binning

Combining both methods which produced Arthropoda annotations, the number of contigs was 3,076 with a genome size of 1,103 MB (1,103,269,596 bp). Results from Kraken2 indicated there was significant bacterial contamination, with very few scaffolds being annotated to other eukaryotes in the Kraken2 binning (Arthropoda: 2,536; Bacteria: 1,413). The Kraken2 binning also revealed a small amount of contamination from microbial eukaryotes (Nematoda: 43, Euglenazoa: 10) (Fig. S2). The BlobTools binning which was quite stringent returned either contigs matching Arthropoda (Arthropoda: 2,830) or undefined ambiguous matches (undef: 780). Both metagenomic binning methods produced a few contigs that did not produce any matches (Kraken2: 63, BlobTools: 460). No contigs were assigned to the Musa genome or fungal genomes. The two methods had a significant amount of overlap with 2,375 or roughly 77% of contigs assigned to Arthropoda shared between methods (Figs. 1, S2 and S3). There was an excellent congruence of 77% between both methods among those classified as Arthropoda with this post-binning approach. See Table 2 for a complete summary of the post-binning assembly statistics.

#### Pre-binning

 Kraken2 classified 99.2% of the HiFi reads with the largest amount, 88.8% to Arthropoda (1,518,858 reads). A small number were assigned to other microbial eukaryotes with 0.63% (10,733) to Nematoda, and 0.19% (3,204) to Euglenozoa and only 0.05% (846) to Musa. Bacteria comprised 8.65% (147,841) of total reads (Fig. S2). BlobTools taxify produced 60,959 (3.55%) and 4,362 (0.26%) reads for Arthropoda and Bacteria respectively. *Taxify* also produced one read for Nematoda and one for Fungi with the 95% reads (1,643,809) being unclassified. A combined pre-binned 1,520,506 reads were passed to Hifiasm for assembly (Fig. S3). The Hifiasm pre-binning assembly produced 3,089 contigs with at total length of 1,071 MB (1,071,277,630 bp). For full summary statistics see Table 2.

### Repeat content

Non-repetitive genomic content comprised roughly 27.4% (282,809,109 bp) and 33.8% (372,152,632 bp) of the pre- and post-binning genome assemblies respectively, compared to repeat content making up the remaining 73.6% (788,468,521 bp) and 66.3% (731,116,964 bp) for pre- and post-binning respectively. Total interspersed repeats comprising 73.6% and 66.3% of repeat content, containing 20.4% and 15.8% LINES, 1.6% and 1.1% LTR elements, 35.5% and 28.9% DNA transposons, with 15.8% and 18.8% unclassified repeats of pre- and post-binning assemblies. The genome also contained Small RNA Retroelements comprising 0.3% and 1.2% of repeats in pre- and post-binning assemblies. For a full list of repeat elements see Table S2.

### Gene prediction

A total of 16,483 and 24,494 genes and 16,483 and 24,740 mRNAs were predicted by AUGUSTUS for pre- and post-binning assemblies. The number of genes with single exon genes was 3,777 and 7,395 for pre- and post-binning assemblies. Multi-exon genes were predicted to be 12,386 and 17,136 for pre- and post-binning assemblies, and the longest gene had a length of 244,960 and 188,748 bp for pre- and post-binning assemblies respectively. A total of 83,512 and 98,982 exons and 67,433 and 74,658 introns were predicted for the pre- and post-binning assemblies respectively. The average exon length was 277 and 307 bp with the average intron length being considerably longer being 1,289 and 1,886 bp for pre- and post-binning assemblies. BUSCO indicated that the AUGUSTUS gene prediction was also complete with 98.6% and 99.0% of genes predicted and 97.1% and 97.0% of them being complete and single copy for pre- and post-binning assemblies (Table 3). For more summary gene prediction data see Table 4.

**Table 3:** Genome completeness of the *Cosompolites sordidus* genome assemblies as measured by BUSCO using the Insecta Odb10 database.

	BUSCO genome mode on pre-metagenomic binning genome	BUSCO genome mode on post-metagenomic binning genome	BUSCO protein mode on pre-metagenomic binning braker2 augustus hints	BUSCO protein mode on post-metagenomic binning braker2 augustus hints
	C:98.6% (S:97.1%, D:1.5%), F:0.7%, M:0.7%, n:1,367	C:99.0% (S:97.%, D:1.4%), F:0.7%, M:0.3%, n:1,367	C:96.3% (S:91.1%, D:5.2%), F:1.1%, M:2.6%, n:1,367	C:92.6% (S:86.6%, D:6.0%), F:1.8%, M:5.6%, n:1,367
Complete BUSCOs (C)	1,348	1,353	1,317	1,266
Complete and single-copy BUSCOs (S)	1,328	1,334	1,246	1,184
Complete and duplicated BUSCOs (D)	20	19	71	82
Fragmented BUSCOs (F)	10	9	15	24
Missing BUSCOs (M)	9	5	35	77
Total BUSCO groups searched	1,367	1,367	1,376	1,367

**Note:**

Table 3: Genome completeness of the *Cosompolites sordidus* genome assemblies as measured by BUSCO using the Insecta Odb10 database.

**Table 4:** *Braker2-AUGUSTUS* gene prediction summary statistics of the *Cosmopolites sordidus* genome with shortest isoform if any dropped out, numbers are the correspond to metagenomic pre-binning/post-binning.

Genomic class	Total	Total length	Longest	Shortest	Mean length	Mean/ gene	Mean length/gene
Gene	16,483/24,494	163,790,859/167,234,975	244,960/188,748	170/166	9,936/6,827	NA	NA
Exon	83,512/98,982	23,173,081/30,402,537	31,523/27,903	3/3	277/307	5.1/4.0	277/307
Intron	67,433/74,658	142,501,905/140,822,402	60,704/46,654	11/11	2,113/1,886	4.1/3.0	2,097/1,869
Coding sequence	16,483/24,740	21,257,275/28,338,732	82,659/82,569	98/24	1,289/1,145	1/1	254/286

### Functional gene annotation

**Figure 2:**
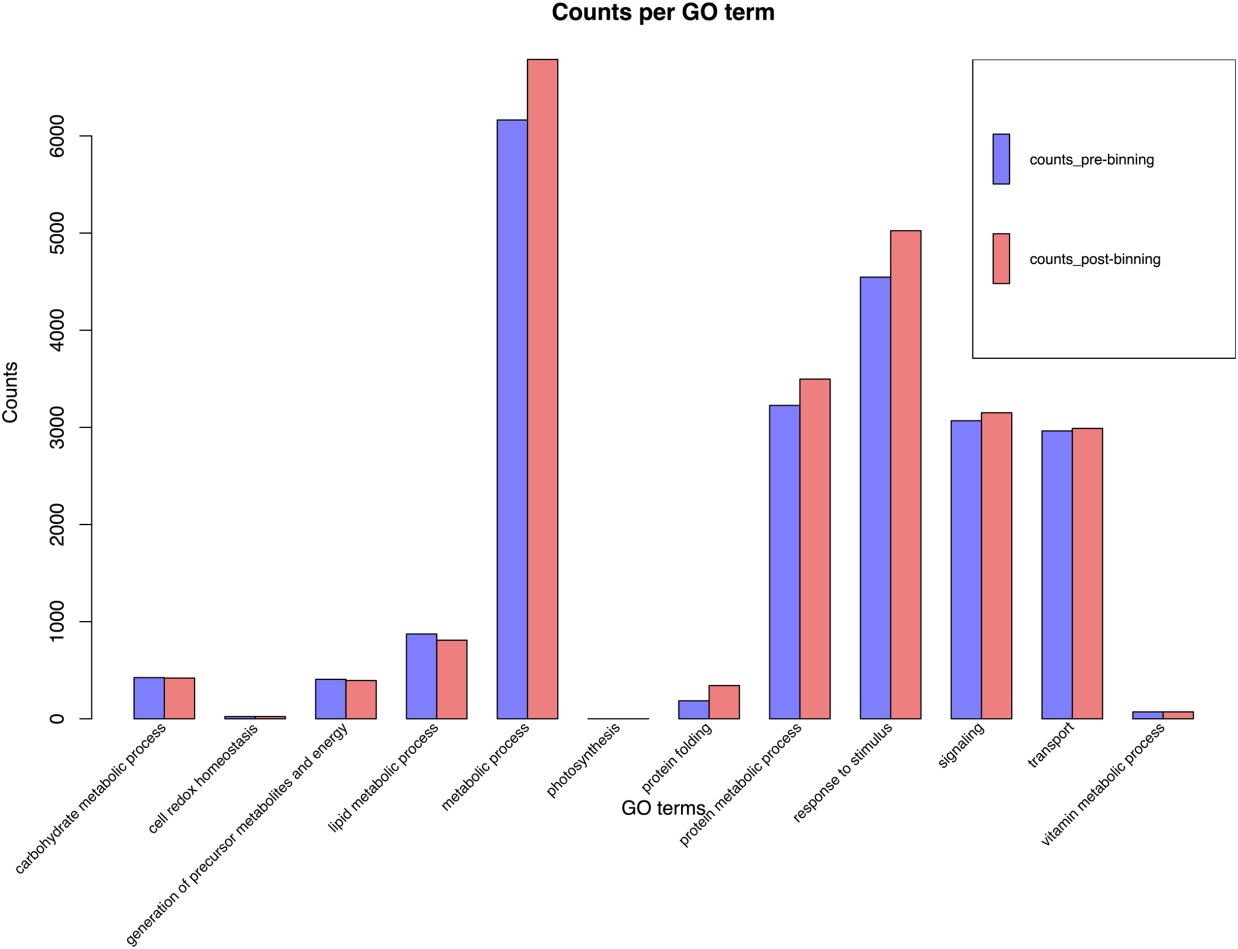
*EggNOG-mapper* histogram plot of major cellular processes derived from the *Cosmopolites sordidus* genome assembly. Genes were predicted *via* Braker2 following the assembly. Pre-binning refers to filtering Arthropoda reads prior to assembly and post-binning refers to binning contigs after the initial assembly to Arthropoda.

**Figure 3:**
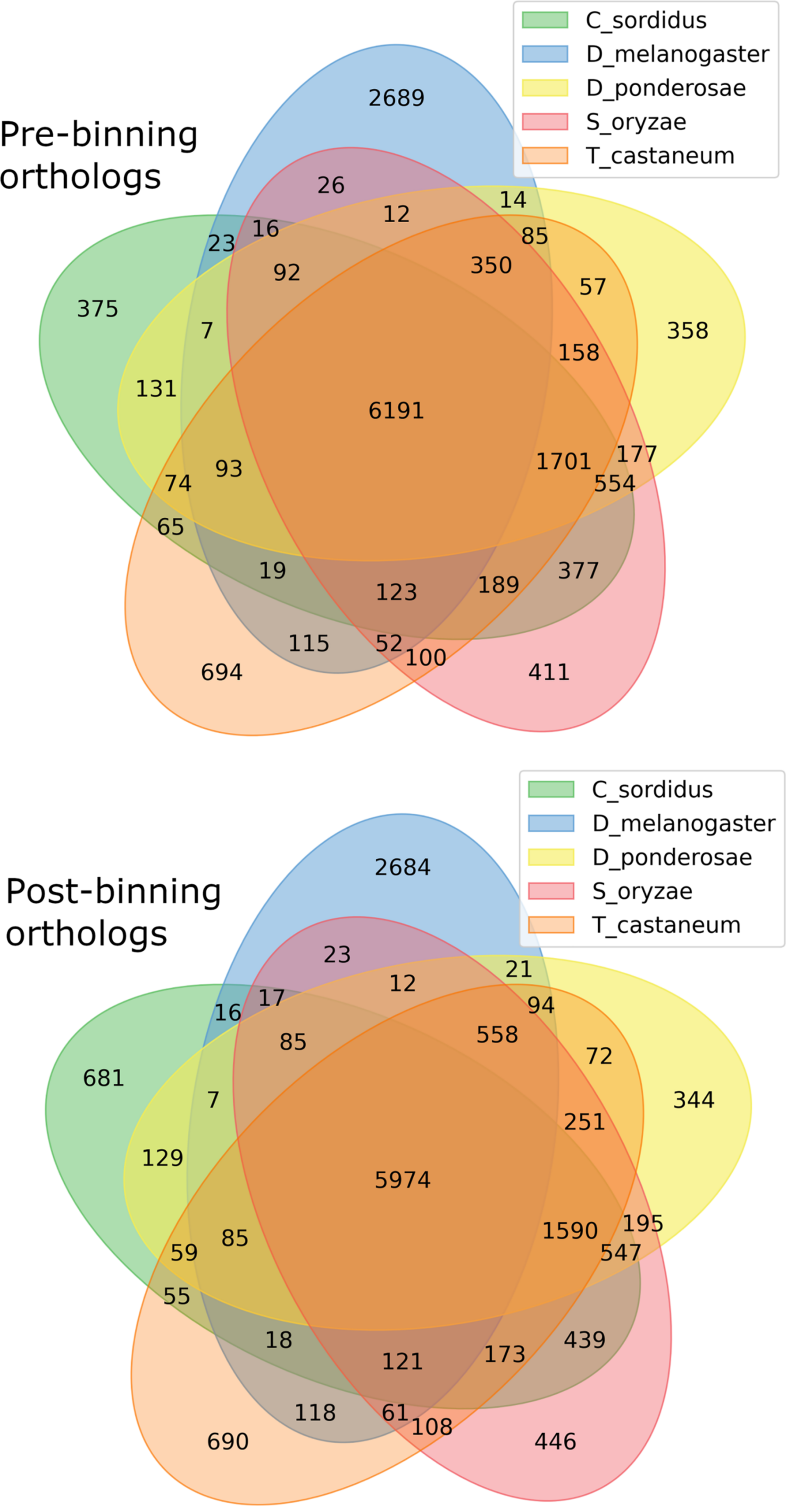
Gene orthology overlap between derived from *OrthoFinder* between the predicted proteins. Pre-binning refers to filtering Arthropoda reads prior to assembly and post-binning refers to binning contigs after the initial assembly to Arthropoda. In both Venn diagrams the order of taxa starting from 12 ’clock proceeding clockwise: *Drosophila melanogaster* (D_melanogaster), *Dendroctonus ponderosae* (D_ponderosae), *Sitophilus oryzae (S_oryzae), Tribolium castaneum* (T_castaneum), and *Cosmopolites sordidus* (C_sordidus).

BUSCO run in protein mode found 96.3% and 92.6% BUSCOs with 91.1% and 86.6% single copy BUSCOs for the pre-and post-binning assemblies respectively. eggNOG-mapper annotated 11,178 and 14,262 or 68% and 58% of genes received annotation using this method for pre- and post-binning assemblies. A summary of major cellular components GO-slims produced *via* egg-NOG-mapper were similar between the two assemblies these results can be found in Fig. 2. Among eggNOG-mapper predictions 179 and 1,158 were associated with Bacteria for the pre- and post-binning assemblies. OrthoFinder assigned a total of 16,648 and 24,740 or 90% and 93% of genes to an orthologous group for the pre-binning and post-binning assemblies respectively. Orthogroup-containing gene species was 2,285 and 8,229 with 13.7% and 33% being unique gene species to the *C. sordidus* genome for the pre- and post-binning assemblies respectively that were not shared by the other four taxa examined. *S. oryzae* shared the most orthogroups in common with *C. sordidus* (9,243 pre- and 8,946 post-binning), with fewer shared in common with increasing taxonomic distance between the three other taxa and *C. sordidus*. For a full summary of OrthoFinder results see Table S3 and Fig. 3.

### Identifying *C. sordidus* coding regions contaminated with HiFi reads of bacterial origins

**Figure 4:**
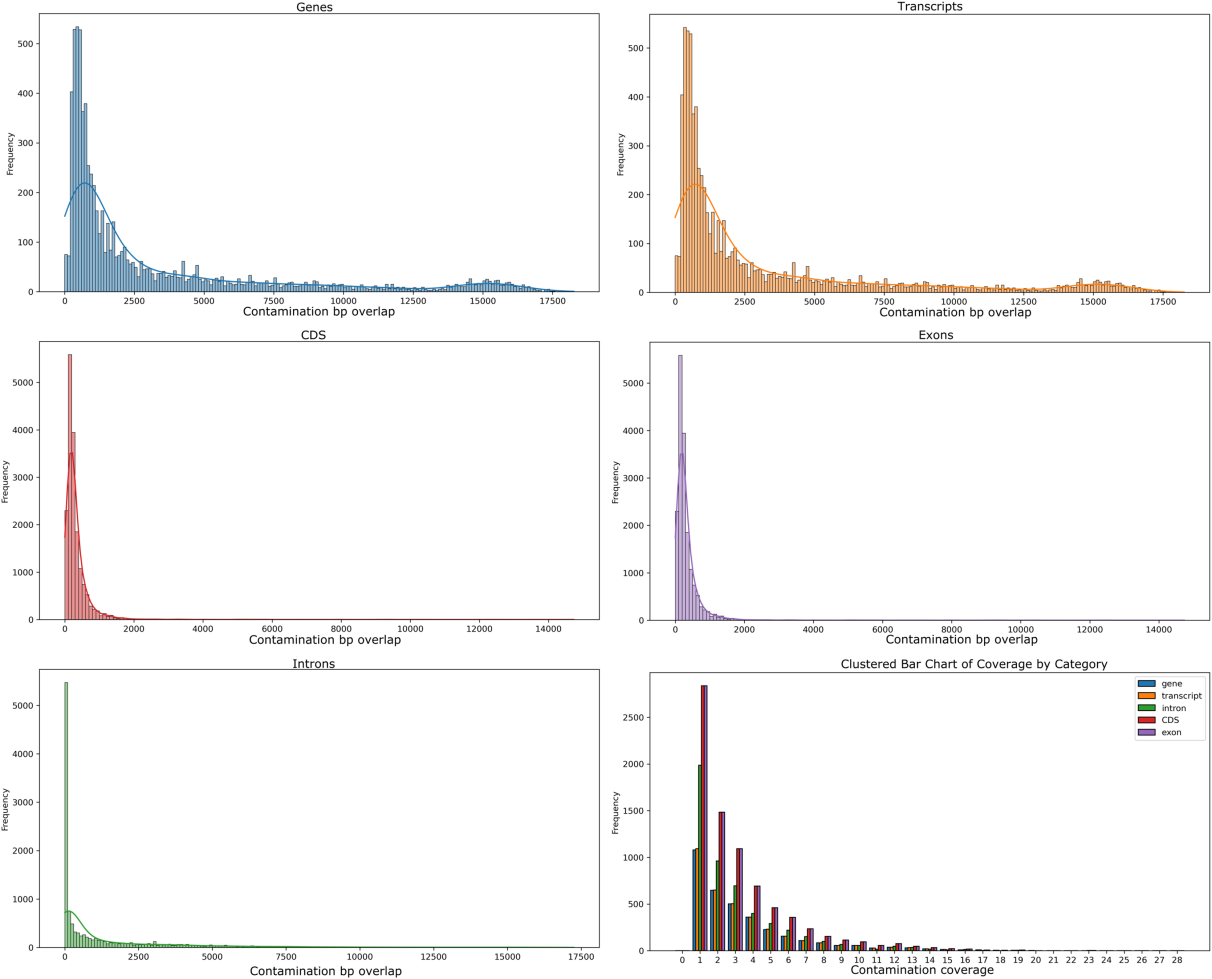
Histograms of putative Bacteria contamination in the *Cosmopolites sordidus* post-binning assembly. The first two rows of histograms and the bottom left histogram refers to the frequency of contamination in predicted intragenic regions of the *C. sordidus* genome that span regions of a given bp size in 10 bp bins. Each histogram is for a different intragenic component. The bottom right histogram is the actual coverage of the contamination only, not including the other overlapping Arthropoda reads found in predicted intragenic components. The bars for the lower right histogram are ordered along the x-axis are ordered as follows: gene, transcript, intron, CDS, and exon.

**Figure 5:**
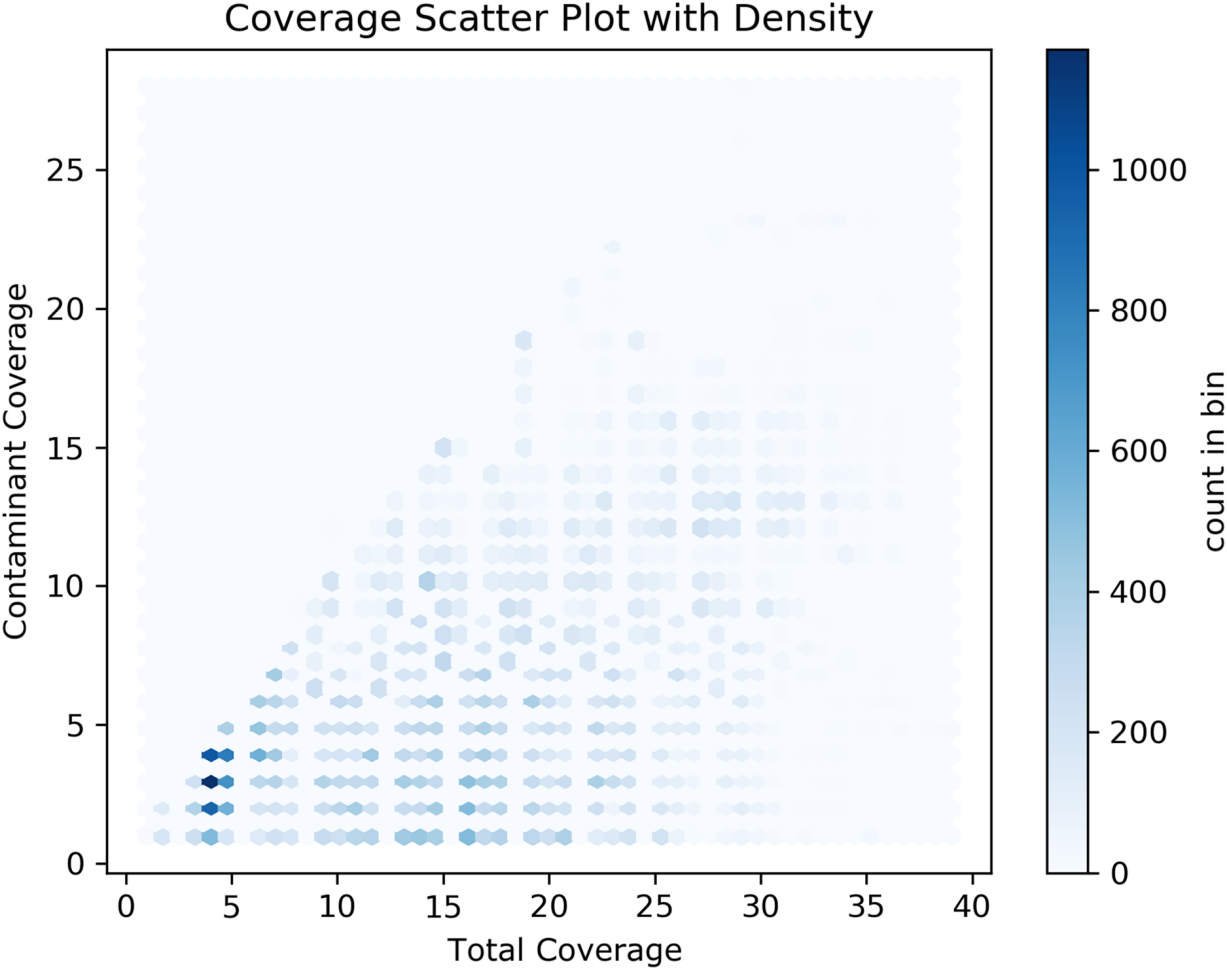
Hexbin scatter plot of the frequency of contamination found in contigs in the post-binning *Cosmopolites sordidus* genome assembly from intragenic regions only. Coverage refers to the total coverage of both Arthropoda and putative Bacteria contaminant read in the predicted intragenic regions of the genome.

Our analyses of the genomic intragenic regions identified 3,433 genes, or 14% of total genes, not only used putative bacterial reads in the assembly and were clearly mapping to regions with the same identity to bacterial reads in the final post-binning assembly. These genes had significant coverage and of base pairs containing putative bacterial contamination (See Fig. 4). Among genes with putative contamination average coverage with standard error per gene was 3.52 ± 0.05, and the average length with standard error of the contamination was 2,675.81 ± 61.02 bp. Total average and standard error coverage for these same regions including all reads was 16.14 ± 0.02. The hexbin plot showed that contained within coding regions of all coverage levels putative Bacteria contamination was present, and it seemed it was most concentrated at lower levels of coverage below 10X. However, there also seemed to b dense outlier bins as well (Fig. 5). The linear regression analyses showed a strong correlation between increasing genomic coverage and increasing putative bacterial contamination coverage (y = 0.25x + 1.83, R^^2^ = 0.21). The logistic regression identified that the frequency of contamination noticed a slight but significant decrease with increasing coverage had an intercept coefficient of −2.6527, and the coefficient for background coverage was −0.0184. This means is that for each unit the log odds of the region being contaminated, compared to not being contaminated, decrease by 0.0184 with a *p*-value close to zero (LLR *p*-value 3.342e–22). For each extra unit of total coverage, the probability of contamination decreases by 0.2%. The results from the linear and logistic regressions should be intuitive from an inspection of the hexbin graph (Fig. 5). Essentially as we increase overall coverage so too does the contamination, but the frequency of the contamination occurring is more likely to occur at lower levels of overall coverage (Fig. 5).

### Final metagenomic contaminant classification using NCBI *FCS-GX*

Using NCBI metagenomic classification program FCS-GX (Astashyn et al., 2023) a final putative contaminant filtering flagged 112 and eight contigs ranging in size from 17–61 kbp and 49–25 kbp at 2,735,043 bp and 268,753 bp total with a mean of 24,420 bp and 33,594.1 bp and standard deviation of 5,930.38 bp and 7,397.71 bp for the pre-and post-binning assemblies respectively. Both assemblies had putative contamination assigned to g-proteobacteria by FCS-GX. The pre-binning assembly was chosen for submission to NCBI (GenBank Genome accession JARFXV000000000) as it has the least amount of contamination throughout the genome. The assemblies for both pre-and post-binning are deposited in the Dryad repository (DOI 10.5061/dryad.f1vhhmh2r) that are associated with the data analyses of this manuscript.

### mtDNA genome annotation

The results from RagTag gave a mtDNA genome read that was 15,386 bp. The *MITOS* (Bernt et al., 2013) annotation revealed that the sequenced mtDNA genome was nearly complete, with only trnI and rrnS not being annotated. All other genes were found, as well as the d-loop region. The cox1 gene from this partial mtDNA genome was aligned to the NCBI nt database using the NCBI blastn webpage (Johnson et al., 2008), the best match was from another cox1 gene from *C. sordidus* (NCBI: AY131111.1) with additional Curculionidae cox1 genes also having significant hits. For a full summary of the MITOS annotation see Table S4. Blat results with significant matches back to the *C. sordidus* contigs can be found in Van Dam et al, 2023, Dryad, https://doi.org/10.5061/dryad.f1vhhmh2r, and the RagTag mtDNA assembly is deposited in the Dryad repository (DOI 10.5061/dryad.f1vhhmh2r).

## Discussion

The results from the HiFi read assembly *via* hifiasm for pre-and post-metagenomic binning indicate a *C. sordidus* genome assembly is that both are high quality in terms of length and completeness. The summary statistics evidence the quality of the assemblies, BUSCO scores both in genome and protein mode, and a number of orthologous genes with the other genomes examined. Both genome assemblies would be very useful in terms of identifying candidate genes for exploring functional genomics. On the other hand, regarding method reliability for studying gene and genome evolution, the pre-binning assembly should be more reliable as it is less likely to contain contamination. Based on the annotation from eggNOG-mapper it appears that the pre-binning genome has significantly fewer protein-coding genes of putative bacterial origins compared to the post-binning genome assembly. In fact, the latter has six and a half times more protein coding genes with a greater affinity to those of bacterial pathways.

It is important to consider that there may be several factors that contribute to the observed difference, such as the varying number of reads used in each assembly. However, we can infer that a significant number of supposed bacterial reads were not only included in the *C. sordidus* post-binning genome assembly but also played a key role in the formation of various protein-coding genes.

There were 8,011 more genes predicted in the post-binning assembly than in the pre-binning approach. This may have carried over to the gene annotation with 3,084 more genes annotated and 5,944 more genes unique to the *C. sordidus* genome annotated by OrthoFinder. While 3,433 genes had bacterial contamination this would not on its own explain the difference in the number of genes predicted and annotated between the two approaches. It may be a combination of factors such as the total number of reads used in the assembly, bacterial contamination, and the effect of having more contigs on the gene prediction process. The present analyses cannot identify a potential cause of this significant effect here. We can, however, make some conclusions about the *C. sordidus* pre-binning genome’s quality and recommendations for future assemblies using HiFi data looking to reduce potential sources of contamination.

In terms of quality, clearly with only 3,089 contigs a genome size of 1.07 Gb with 98.6% and 97.1% complete and single copy BUSCO scores for genome and protein modes respectively the pre-binning assembly is by all accounts very high quality. With only a small difference in total genome size and greater completeness regarding BUSCO protein annotation, the pre-binning approach delivers a slightly smaller yet slightly higher quality genome. The pre-binning approach in this case does not make sacrifices of genome completeness while eliminating many more potential sources of contamination. What we are left with is a genome that is of high quality and of standards that can be used for studying functional genomics as well as genome evolution.

The analyses of putative contamination making its way into the *C. sordidus* post-binning genome make it clear that potential sources of contamination were throughout this assembly. This was especially true of the low coverage contigs. The present study is a sample size of one, and it would be very interesting to investigate the recent assemblies that also used post-binning as an assembly strategy. For example in there are 158 HiFi genomes in the Ag100Pest Initiative (Childers et al., 2021) and the Darwin Tree of Life project has 2,696 proposed genomes so far, both projects use post-binning for their metagenomics strategy (The Python Venn Developers, 2022; Falk & Monks, 2022; Falk & Broad, 2022; Boyes & Lewin, 2022). Both of these large research groups use additional data such as RNA-seq (Childers et al., 2021) and 10X Chromium sequencing (Falk & Monks, 2022), these methods would provide a means to validate the HiFi data. The RNA-seq data as it is poly-A tailed can be used to invalidate false positives for contamination and the 10X Chromium sequencing adds additional coverage to prevent contamination. However, as the contamination that we are examining in the HiFi assembly process with the *C. sordidus* genome occurs before the addition of other data types it would still not eliminate contamination that is interspersed within the contigs as we find here. It is very likely that do to the high coverage in both the Ag100Pest and Darwin Tree of Life projects that all the additional coverage would completely swamp out contamination. On the other hand, for smaller projects that do not have a similarly sized budget to include additional coverage, pre-binning of HiFi reads should be an essential consideration.

Arthropods have open circulatory systems. In a previous study it was found that using arthropod muscle tissue alone does not completely eliminate Bacteria or microbial Eukaryote contamination from being carried on downstream to the assembly process (Van Dam et al., 2022). The widespread methodology utilized by the community seems to assume that long-reads tend to eliminate misincorporation of contaminants. Based on the evidence in the present study high quality long-reads do not seem to prevent misincorporation of contamination in arthropod genome assembly. Obviously, the present study is a sample size of one, and while the analyses here were thorough, similar methods should be replicated many more times on a larger more diverse data set of arthropods.

PacBio HiFi sequencing is an extremely powerful technique and has played a central role in the present study and much larger sequencing efforts such as the Ag100Pest and Darwin Tree of Life initiatives. HiFi sequencing is also relatively new at the time of this article preparation it will be less than 4 years since its announcement (Wenger et al., 2019). Post-binning reference based metagenomic approaches have been widely used for eukaryotic genome assemblies for this data type. We present here a much less commonly use but practical reference-based pre-binning approach for HiFi data. One of the significant problems with referenced based approaches is that they are only as good as the reference database being used. So far only one entirely reference free pre-binning based approach, LRBinner, has been developed (Wickramarachchi & Lin, 2022). However it has only been tested on Bacteria metagenomic assemblies (Wickramarachchi & Lin, 2022; Ibañez-Lligoña et al., 2023). A combination of using LRBinner (Wickramarachchi & Lin, 2022) along with RNA-seq based scaffolding (Xue et al., 2013) and Illumina paired end data pre-binned *via* reference free binning *eg.* AAMB or VAMB (Nissen et al., 2021; Piera Lindez et al., 2023) could be a way forward to remove contamination in HiFi data before assembly. Adding Hi-C data would only further improve the assembly but not remove interspersed contamination. Hi-C data has been shown to improve assemblies significantly even in highly repetitive Curculionidae genomes like *C. sordidus* (Van Dam et al., 2021).

The final metagenomic filtering pass with FCS-GX indicates that both pre- and post-binning metagenomic techniques are required to remove as much contamination as possible from genome assemblies that utilize PacBio HiFi data alone to assemble arthropod genomes. In the pre-binning assembly, 112 contigs were identified as having contamination by FCS-GX. This is an exciting result suggesting that this novel hybrid h-mer k-mer alignment-based approach may be more sensitive to contamination than the k-mer-based alignment-free approach that we primarily used to bin out contamination from our pre-binning assembly using Kraken2. However, the FCS-GX manuscript does not directly compare this new method to existing k-mer based methods for metagenomic binning and classification, making it difficult to discern how this new method compares to other existing k-mer-based approaches or hybrid h-mer k-mer programs like FCS-GX (Astashyn et al., 2023). Additionally, the FCS-GX paper does not investigate the degree of accuracy of its predictions as the evolutionary distance increases from the nearest sister species included in the database making it difficult to identify the probability of miss-annotations in genome assemblies of novel clades (Astashyn et al., 2023). NCBI released the FCS-GX paper in June of 2023 after the main analyses for this project were already completed. Otherwise it would have certainly been interesting to include it here as both a pre- and post-binning approach. FCS-GX is utilized as a post-binning approach, and it failed to identify contaminated regions in the post-binning assembly that the present investigation identified using the pre-binning data. All that said FCS-GX is a very promising approach for binning PacBio HiFi data as it seems to combine the speed of k-mer-based approaches with the accuracy of alignment based metagenomic classification programs.

Finally, we hope that the *C. sordidus* genome assembly presented here will be of use to scientists interested in the evolution of K-selected arthropods, Curculionoidea evolution, and those interested in developing gene-drive technology or other IPM strategies for this significant economic pest.

## Supplemental Information

10.7717/peerj.16276/supp-1 Supplemental Information 1 Outline of the overall assembly and genome annotation process for the *Cosmopolites sordidus* genome.

10.7717/peerj.16276/supp-2 Supplemental Information 2 Kraken2 results plotted using *krona* for the *Cosmopolites sordidus* genome assemblies.

10.7717/peerj.16276/supp-3 Supplemental Information 3 Euler Venn diagrams of the *Cosmopolites sordidus* genome assemblies. Top, overlap of Arthropoda contigs in the post-binning assembly by methods, left circle Kraken2 and right circle BlobTools. Middle, overlap of Arthropoda HiFi reads used as input in the pre-binning assembly by methods, left circle Kraken2 and right circle BlobTools. Bottom, orthogroup overlap as per *OrthroFinder* between the pre-binning assembly (left) and post-binning assembly (right).

10.7717/peerj.16276/supp-4 Supplemental Information 4 Eukaryotic genomes and their accession numbers used for metagnomic binning of the *Cosmopolites sordidus* using hifiasm assembly program.

10.7717/peerj.16276/supp-5 Supplemental Information 5 *RepeatMasker* results from the *Cosmopolites sordidus* genome assemblies.

10.7717/peerj.16276/supp-6 Supplemental Information 6 *OrthoFinder* results comparing predicted proteins from NCBI RefSeq genomes and predicted proteins from the *Cosmopolites sordidus* genome, with pre-metagenomic filtering results followed by a “ / ” and the post-metagenomic binning results. Abbreviations: from left to right *Cosmopolites sordidus*, *Drosophila melanogaster*, *Triboliums castanemum*, *Dendroctonus ponderosae*, and *Sitophilus oryzae*.

10.7717/peerj.16276/supp-7 Supplemental Information 7 MITOS *Cosmopolites sordidus* mtDNA genome *RagTag* assembly annotation and gene prediction.

## Additional Information and Declarations

### Data Availability

The following information was supplied regarding data availability: The data is available at NCBI SRA: SRR18555109 and PRJNA817621.
