# Supplementary figures and images for: Metagenomic binning of PacBio HiFi data prior to assembly reveals a complete genome of Cosmopolites sordidus (Germar) (Coleopterea: Curculionidae, Dryophthorinae) the most damaging arthropod pest of bananas and plantains
Source: PeerJ. 2023 Nov 22;11:e16276. doi: 10.7717/peerj.16276 (PMC10676084; doi:10.7717/peerj.16276)

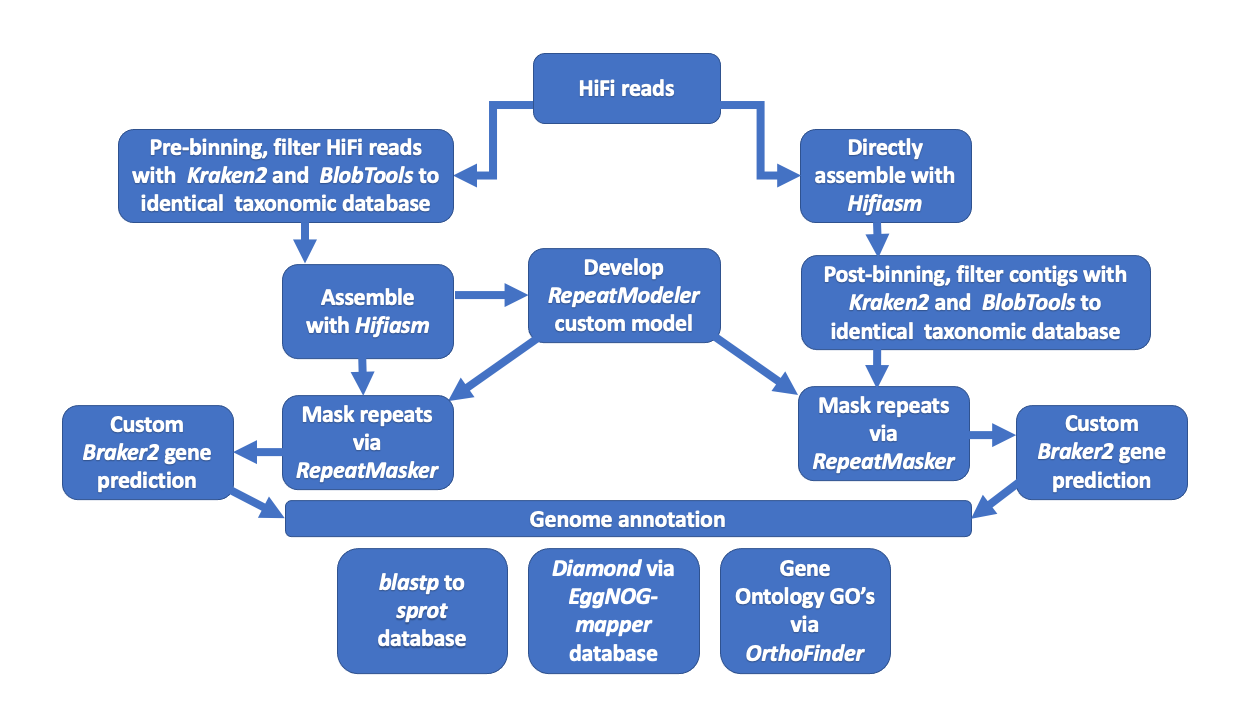

Supplement: Supplemental Information 1 [file peerj-11-16276-s001.png]

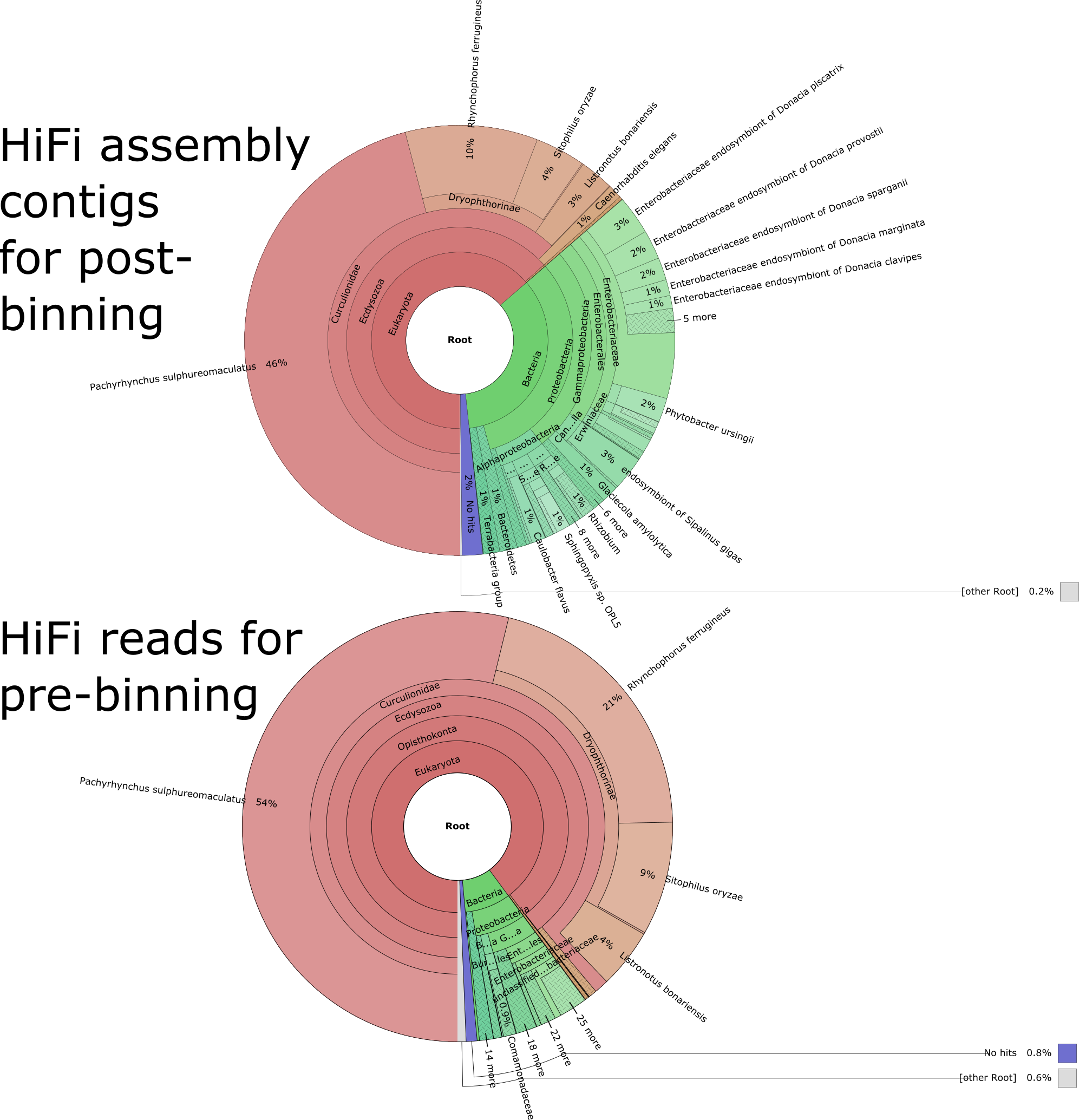

Supplement: Supplemental Information 2 [file peerj-11-16276-s002.png]

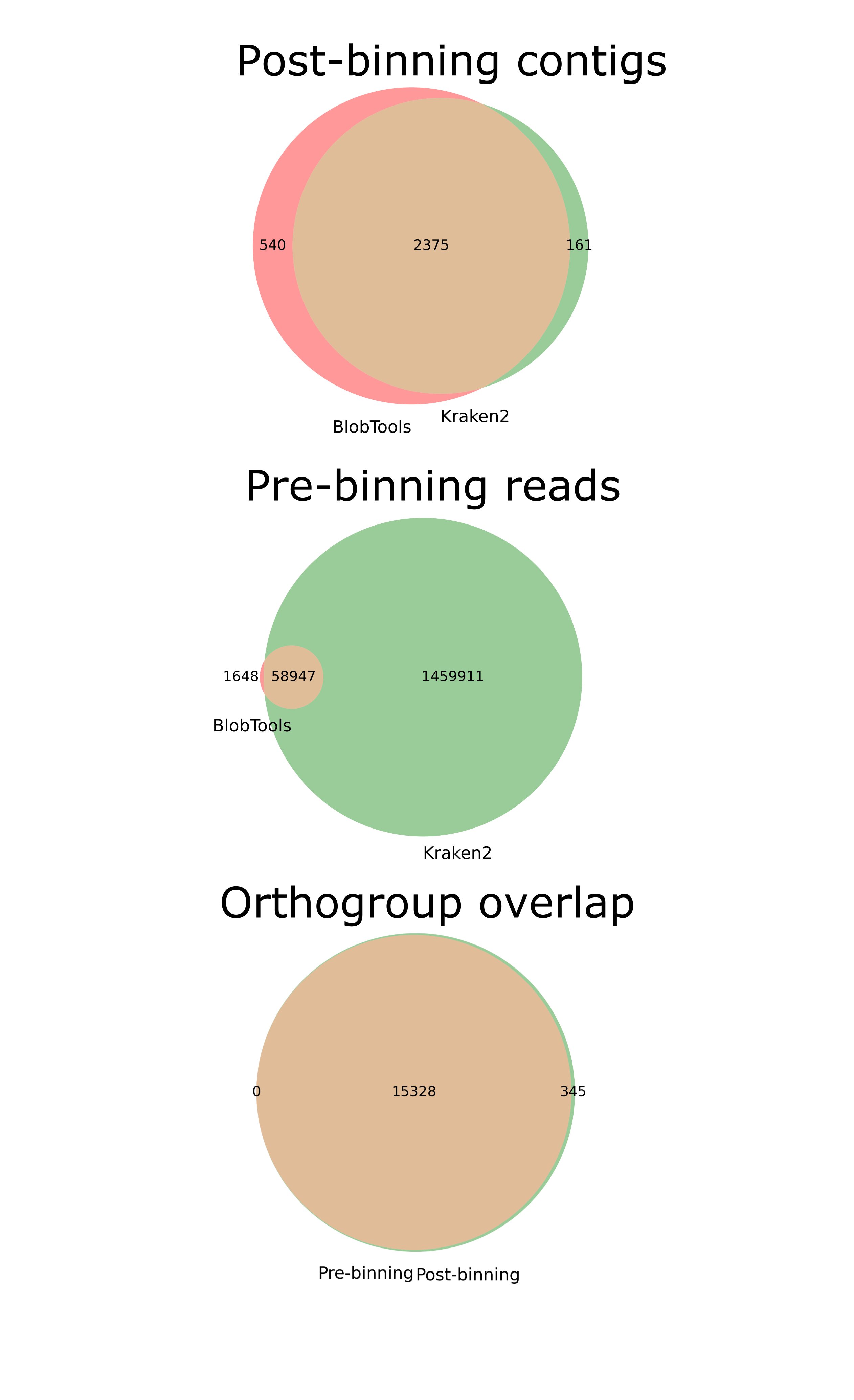

Supplement: Supplemental Information 3 — Top, overlap of Arthropoda contigs in the post-binning assembly by methods, left circle Kraken2 and right circle BlobTools. Middle, overlap of Arthropoda HiFi reads used as input in the pre-binning assembly by methods, left circle Kraken2 and right circle BlobTools. Bottom, orthogroup overlap as per OrthroFinder between the pre-binning assembly (left) and post-binning assembly (right). [file peerj-11-16276-s003.png]
