# Supplementary material for: Metagenomic binning of PacBio HiFi data prior to assembly reveals a complete genome of Cosmopolites sordidus (Germar) (Coleopterea: Curculionidae, Dryophthorinae) the most damaging arthropod pest of bananas and plantains
Source: PeerJ. 2023 Nov 22;11:e16276. doi: 10.7717/peerj.16276 (PMC10676084; doi:10.7717/peerj.16276)
Supplement: Supplemental Information 4 [file peerj-11-16276-s004.docx]

**Table S1**: Eukaryotic genomes and their accession numbers used for metagnomic binning of the *Cosmopolites sordidus* hifiasm assembly.

| **Custom Metagenomic Database Genomes** | |
| --- | --- |
| **Repository** | **Accession** |
| Kraken2 | kraken2-build --download-library bacteria |
| NCBI | GCA_000002985.3_WBcel235_genomic.fna.gz |
| NCBI | GCA_000146045.2_R64_genomic.fna.gz |
| NCBI | GCA_000280035.2_ASM28003v2_genomic.fna.gz |
| NCBI | GCA_000300575.2_ASM30057v2_genomic.fna.gz |
| NCBI | GCA_000313855.2_ASM31385v2_genomic.fna.gz |
| NCBI | GCA_000388065.2_Font_alba_ATCC_38817_V2_genomic.fna.gz |
| NCBI | GCA_002102555.1_Catan2_genomic.fna.gz |
| NCBI | GCA_002214945.1_ASM221494v1_genomic.fna.gz |
| NCBI | GCA_002938485.2_Soryzae_2.0_genomic.fna.gz |
| NCBI | GCA_003325435.1_Razy_CA_genomic.fna.gz |
| NCBI | GCA_004359215.2_BlacSF5v2_genomic.fna.gz |
| NCBI | GCA_012979105.1_ASM1297910v1_genomic.fna.gz |
| NCBI | GCA_014170235.1_ASM1417023v1_genomic.fna.gz |
| NCBI | GCA_015099795.1_ASM1509979v1_genomic.fna.gz |
| NCBI | GCA_019049505.1_P_sulf_FINAL_HiC_nocotam_named_genomic_k2.fna.gz |
| NCBI | GCA_900093555.2_GCA_900093555_genomic.fna.gz_k2.fna.gz |
| NCBI | GCA_900404475.1_SSUBK13_genomic.fna.gz_k2.fna.gz |
| NCBI | GCA_900893395.1_EugGra_PACBIO_Illumina_merge_V1_genomic.fna.gz_k2.fna.gz |
| NCBI | GCA_910592215.1_Claroideoglomus_candidum_CCK_pot_B_6-9_genomic.fna.gz |
| NCBI | GCF_000002435.2_UU_WB_2.1_genomic.fna.gz_k2.fna.gz |
