# Supplementary material for: Metagenomic binning of PacBio HiFi data prior to assembly reveals a complete genome of Cosmopolites sordidus (Germar) (Coleopterea: Curculionidae, Dryophthorinae) the most damaging arthropod pest of bananas and plantains
Source: PeerJ. 2023 Nov 22;11:e16276. doi: 10.7717/peerj.16276 (PMC10676084; doi:10.7717/peerj.16276)
Supplement: Supplemental Information 5 [file peerj-11-16276-s005.docx]

**Table S2**: *RepeatMasker* results from the *Cosmopolites sordidus* genome assemblies.

| **RepeatMasker Repeat Content** | Pre-binning / Post-binning | | |
| --- | --- | --- | --- |
|  | Repeat number | Number of base pairs (bp) | Percent of repeats |
| Retroelements | 530354 / 569555 | 232063553 / 185963452 bp | 21.66% / 16.8% |
| SINEs: | 0 / 285 | 0 / 28520 | 0 / 0 |
| Penelope | 189441 / 244964 | 48922932 bp / 58267585 bp | 4.57% / 5.28% |
| LINEs: | 508532 / 557505 | 214653404 bp / 174141109 bp | 20.04% / 15.78% |
| CRE/SLACS | 0 / 0 | 0 / 0 | 0.00%/ 0.00% |
| L2/CR1/Rex | 25511 / 36869 | 13403098 bp / 12914926 bp | 1.25% / 1.17% |
| R1/LOA/Jockey | 30573 / 19247 | 10587637 bp / 11243674 bp | 0.99% / 1.02% |
| R2/R4/NeSL | 22711 / 22823 | 17697349 bp / 17180609 bp | 1.65% / 1.56% |
| RTE/Bov-B | 203448 / 205513 | 103538303 bp / 60532813 bp | 9.66% / 5.49% |
| L1/CIN4 | 202 / 0 | 10355 bp / 0bp | >0.00% / 0.00% |
| LTR elements: | 21822 / 11765 | 17410149 bp / 11793823 bp | 1.63% / 1.07% |
| BEL/Pao | 1336 / 626 | 1481277 bp / 725818 bp | 0.14% / 0.07% |
| Ty1/Copia | 1452 / 1548 | 874161 bp / 949436 bp | 0.08% / 0.09% |
| Gypsy/DIRS1 | 14061 / 9591 | 14105336 bp 10118569 bp | 1.32% / 0.92% |
| Retroviral | 4568 / 0 | 297914 bp / 0 | 0.03% / 0.00% |
| DNA transposons | 1401833 / 1066648 | 379889721 bp / 319237153 bp | 35.46% / 28.94% |
| hobo-Activator | 55211 / 56984 | 23155253 bp / 20632782 bp | 2.16% / 1.87% |
| Tc1-IS630-Pogo | 969190 / 900160 | 281742362 bp / 258392266 bp | 26.3% / 23.42% |
| En-Spm | 0 / 0 | 0 / 0 | 0%/ 0% |
| MuDR-IS905 | 0 / 0 | 0 / 0 | 0% / 0% |
| PiggyBac | 30703 / 16796 | 13552956 bp / 7373671 bp | 1.27% / 0.67% |
| Tourist/Harbinge | 6504 / 4949 | 2091446 bp / 1718546 bp | 0.2% / 0.16% |
| Other (Mirage, P-element, Transib) | 20361 / 2675 | 3897051 bp / 1423538 bp | 0.36% / 0.13% |
| Rolling-circles | 24878 / 19814 | 4739958 bp / 3878691 bp | 0.44% / 0.35% |
| Unclassified: | 828671 / 912308 | 169139288 bp / 207225359 bp | 15.79% / 18.78% |
| Total interspersed repeats |  | 781092562 bp / 712425964 | 72.91% / 64.57% |
| Small RNA: | 7106 / 40680 | 2636001 bp / 13684433 bp | 0.25% / 1.24% |
| Satellites: | 0 | 0 | 0% |
| Simple repeats: | 0 / 21910 | 0 / 1127876 bp | 0% / 0.10% |
| Low complexity: | 0 | 0 | 0% |
