# Supplementary material for: Metagenomic binning of PacBio HiFi data prior to assembly reveals a complete genome of Cosmopolites sordidus (Germar) (Coleopterea: Curculionidae, Dryophthorinae) the most damaging arthropod pest of bananas and plantains
Source: PeerJ. 2023 Nov 22;11:e16276. doi: 10.7717/peerj.16276 (PMC10676084; doi:10.7717/peerj.16276)
Supplement: Supplemental Information 6 — Abbreviations: from left to right Cosmopolites sordidus, Drosophila melanogaster, Triboliums castanemum, Dendroctonus ponderosae, and Sitophilus oryzae. [file peerj-11-16276-s006.docx]

**Table S3:** OrthoFinder Results comparing predicted proteins from NCBI RefSeq genomes and predicted proteins from the *Cosmopolites sordidus* genome, with pre-metagenomic filtinerg results followed by a “ / “ and the post-metagenomic binning results. Abbreviations: from left to right *Cosmopolites sordidus*, *Drosophila melanogaster*, *Triboliums castanemum*, *Dendroctonus ponderosae*, and *Sitophilus oryzae*.

| **Statistics Per Species** | *C_sordidus* | *D_melanogaster* | *T_castaneum* | *Dend_ponderosae* | *S_oryzae* |
| --- | --- | --- | --- | --- | --- |
| Number of genes | 16648 / 24740 | 30717 / 30717 | 22610 / 22610 | 20551 / 20551 | 23498 / 23948 |
| Number of genes in orthogroups | 14978 / 23001 | 28059 / 28111 | 21696 / 21702 | 19872 / 19869 | 22931 / 22915 |
| Number of unassigned genes | 1670 / 1739 | 2658 / 2606 | 914 / 908 | 679 / 682 | 567 / 583 |
| Percentage of genes in orthogroups | 90.0 / 93.0 | 91.3 / 91.5 | 96 / 96 | 96.7 / 96.7 | 97.6 / 97.5 |
| Percentage of unassigned genes | 10.0 / 7.0 | 8.7 / 8.5 | 4.0 /4.0 | 3.3 / 3.3 | 2.4 / 2.5 |
| Number of orthogroups containing species | 10030 / 9996 | 9907 /9894 | 10066 / 10027 | 10054 / 10023 | 10529 / 10600 |
| Percentage of orthogroups containing species | 65.4 / 63.8 | 64.6 / 63.1 | 65.7 / 64.0 | 65.6 / 64.0 | 68.7 / 67.6 |
| Number of species-specific orthogroups | 375 / 681 | 2689 / 2684 | 694 / 690 | 358 / 344 | 411 / 446 |
| Number of genes in species-specific orthogroups | 2285 / 8229 | 9888 / 9839 | 2921 / 2942 | 1082 / 1065 | 2100 / 2179 |
| Percentage of genes in species-specific orthogroups | 13.7 / 33.3 | 32.2 / 32.2 | 12.9 / 13.0 | 5.3 / 5.2 | 8.9 / 9.3 |
| **Orthogroups Overlap** |  |  |  |  |  |
| *C_sordidus* | 10030 / 9996 | 6564 / 6323 | 8455 / 8075 | 8873 / 8476 | 9243 / 8946 |
| *D_melanogaster* | 6564 / 6323 | 9907 / 9894 | 7028 / 7029 | 6844 / 6836 | 6862 / 6851 |
| *T_castaneum* | 8455 / 8075 | 7028 / 7029 | 10066 / 10027 | 8709 / 8683 | 8864 / 8836 |
| *Dend_ponderosae* | 8843 / 8476 | 6844 / 6836 | 8709 / 8683 | 10054 / 10023 | 9235 / 9212 |
| *S_oryzae* | 9243 / 8946 | 6862 / 6851 | 8864 / 8836 | 9235 / 9212 | 10529 / 10600 |
