# Supplementary material for: Metagenomic binning of PacBio HiFi data prior to assembly reveals a complete genome of Cosmopolites sordidus (Germar) (Coleopterea: Curculionidae, Dryophthorinae) the most damaging arthropod pest of bananas and plantains
Source: PeerJ. 2023 Nov 22;11:e16276. doi: 10.7717/peerj.16276 (PMC10676084; doi:10.7717/peerj.16276)
Supplement: Supplemental Information 7 [file peerj-11-16276-s007.docx]

**Table S4.** MITOS *Cosmopolites sordidus* mtDNA genome *RagTag* assembly annotation and gene prediction.

| Assembley | start | stop | product | score |  |
| --- | --- | --- | --- | --- | --- |
| C_sordidus_mtDNA_genome_RagTag | 283 | 352 | trnQ(caa) | 1.63E-07 | - |
| C_sordidus_mtDNA_genome_RagTag | 351 | 420 | trnM(atg) | 2.32E-11 | + |
| C_sordidus_mtDNA_genome_RagTag | 438 | 822 | nad2_a | 27619644.7 | + |
| C_sordidus_mtDNA_genome_RagTag | 808 | 1387 | nad2_b | 40175046.1 | + |
| C_sordidus_mtDNA_genome_RagTag | 1427 | 1493 | trnW(tga) | 7.54E-12 | + |
| C_sordidus_mtDNA_genome_RagTag | 1492 | 1556 | trnC(tgc) | 2.14E-08 | - |
| C_sordidus_mtDNA_genome_RagTag | 1556 | 1619 | trnY(tac) | 2.55E-09 | - |
| C_sordidus_mtDNA_genome_RagTag | 1611 | 3132 | cox1 | 286549148 | + |
| C_sordidus_mtDNA_genome_RagTag | 3151 | 3216 | trnL2(tta) | 2.85E-10 | + |
| C_sordidus_mtDNA_genome_RagTag | 3237 | 3882 | cox2 | 105002999 | + |
| C_sordidus_mtDNA_genome_RagTag | 3904 | 3974 | trnK(aag) | 1.16E-06 | + |
| C_sordidus_mtDNA_genome_RagTag | 3974 | 4038 | trnD(gac) | 1.20E-08 | + |
| C_sordidus_mtDNA_genome_RagTag | 4038 | 4143 | atp8-0 | 100594.7 | + |
| C_sordidus_mtDNA_genome_RagTag | 4135 | 4192 | atp8-1 | 3172.1 | + |
| C_sordidus_mtDNA_genome_RagTag | 4188 | 4353 | atp6-1 | 2260872.5 | + |
| C_sordidus_mtDNA_genome_RagTag | 4363 | 4852 | atp6-0 | 33450596.2 | + |
| C_sordidus_mtDNA_genome_RagTag | 4892 | 5084 | cox3-0_a | 27356816.7 | + |
| C_sordidus_mtDNA_genome_RagTag | 5064 | 5649 | cox3-0_b | 102334371 | + |
| C_sordidus_mtDNA_genome_RagTag | 5660 | 5724 | trnG(gga) | 1.26E-08 | + |
| C_sordidus_mtDNA_genome_RagTag | 5733 | 5946 | nad3_a | 6904504.4 | + |
| C_sordidus_mtDNA_genome_RagTag | 5941 | 6067 | nad3_b | 3270772.8 | + |
| C_sordidus_mtDNA_genome_RagTag | 6074 | 6140 | trnA(gca) | 4.89E-08 | + |
| C_sordidus_mtDNA_genome_RagTag | 6140 | 6206 | trnR(cga) | 3.56E-08 | + |
| C_sordidus_mtDNA_genome_RagTag | 6206 | 6274 | trnN(aac) | 7.40E-08 | + |
| C_sordidus_mtDNA_genome_RagTag | 6274 | 6342 | trnS1(aga) | 3.86E-07 | + |
| C_sordidus_mtDNA_genome_RagTag | 6342 | 6411 | trnE(gaa) | 2.16E-07 | + |
| C_sordidus_mtDNA_genome_RagTag | 6411 | 6477 | trnF(ttc) | 2.63E-08 | - |
| C_sordidus_mtDNA_genome_RagTag | 6478 | 8155 | nad5 | 269286131 | - |
| C_sordidus_mtDNA_genome_RagTag | 8188 | 8253 | trnH(cac) | 3.74E-08 | - |
| C_sordidus_mtDNA_genome_RagTag | 8258 | 9536 | nad4 | 230591084 | - |
| C_sordidus_mtDNA_genome_RagTag | 9583 | 9799 | nad4l | 2818975.2 | - |
| C_sordidus_mtDNA_genome_RagTag | 9865 | 9936 | trnT(aca) | 4.23E-06 | + |
| C_sordidus_mtDNA_genome_RagTag | 9936 | 10001 | trnP(cca) | 1.32E-10 | - |
| C_sordidus_mtDNA_genome_RagTag | 10006 | 10471 | nad6 | 3036299.4 | + |
| C_sordidus_mtDNA_genome_RagTag | 10526 | 11606 | cob | 308655131 | + |
| C_sordidus_mtDNA_genome_RagTag | 11652 | 11719 | trnS2(tca) | 4.30E-07 | + |
| C_sordidus_mtDNA_genome_RagTag | 12991 | 13204 | nad1_c | 23357219.7 | - |
| C_sordidus_mtDNA_genome_RagTag | 13196 | 13667 | nad1_b | 55522616.3 | - |
| C_sordidus_mtDNA_genome_RagTag | 13677 | 13854 | nad1_a | 13648001 | - |
| C_sordidus_mtDNA_genome_RagTag | 13896 | 13964 | trnL1(cta) | 1.98E-07 | - |
| C_sordidus_mtDNA_genome_RagTag | 13910 | 15282 | rrnL | 1.13E-11 | - |
| C_sordidus_mtDNA_genome_RagTag | 15290 | 15355 | trnV(gta) | 1.99E-07 | - |
